# Spatial-temporal graph neural ODE networks for skeleton-based action recognition

**DOI:** 10.1038/s41598-024-58190-9

**Published:** 2024-04-01

**Authors:** Longji Pan, Jianguang Lu, Xianghong Tang

**Affiliations:** Guizhou University, State Key Laboratory of Public Big Data, Guiyang, 550025 China

**Keywords:** Computational science, Computer science, Information technology, Software

## Abstract

In the field of skeleton-based action recognition, accurately recognizing human actions is crucial for applications such as virtual reality and motion analysis. However, this task faces challenges such intraindividual action differences and long-term temporal dependencies. To address these challenges, we propose an innovative model called spatial-temporal graph neural ordinary differential equations (STG-NODE). First, in the data preprocessing stage, the dynamic time warping (DTW) algorithm is used to normalize and calculate 3D skeleton data to facilitate the derivation of customized adjacency matrices for improving the influence of intraindividual action differences. Secondly, a custom ordinary differential equation (ODE) integrator is applied based on the initial conditions of the temporal features, producing a solution function that simulates the dynamic evolution trend of the events of interest. Finally, the outstanding ODE solver is used to numerically solve the time features based on the solution function to increase the influence of long-term dependencies on the recognition accuracy of the model and provide it with a more powerful temporal modeling ability. Through extensive experiments conducted on the NTU RGB+D 60 and Kinetics Skeleton 400 benchmark datasets, we demonstrate the superior performance of STG-NODE in the action recognition domain. The success of the STG-NODE model also provides new ideas and methods for the future development of the action recognition field.

## Introduction

Rapid advancements within the field of computer vision have had profound and far-reaching impacts across various domains^1–3^. Within this realm, action recognition stands as a pivotal branch, that is dedicated to the comprehension and analysis of human actions in images and videos. However, to further improve the robustness and practicality of recognition, the field of skeleton-based action recognition is emerging. Within this subfield, traditional methods rely on red-green-blue (RGB) data^4,5^; in contrast, skeletal data encompass time series that encapsulate the 2D or 3D positional coordinates of multiple human joints. These data can be directly captured by sensor devices or extracted from images using pose estimation techniques. Compared with conventional RGB video recognition approaches, action recognition based on skeleton data demonstrates reduced sensitivity to disruptive factors such as changes in lighting, environmental backgrounds, and occlusions that occur during the recognition process. This resilience to environmental variations enhances the robustness and practicality of action recognition systems, making them more reliable across a range of real-world scenarios. Notably, skeleton-based action recognition technology offers potent solutions in various applications, encompassing video surveillance, human-computer interaction, and video comprehension, among others^6,7^. It is an efficient, noninvasive^8^ and robust recognition method and is indispensable in the field of computer vision.

**Figure 1:**
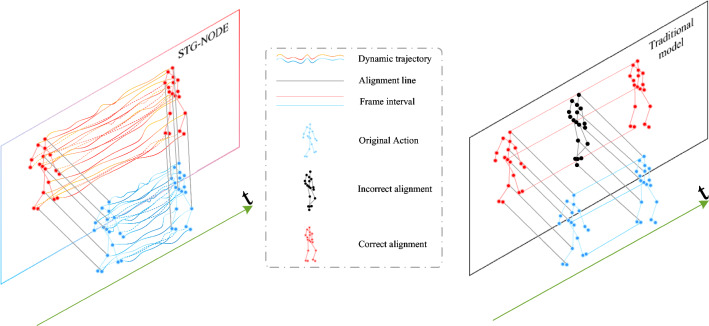
The main idea of this work. Most traditional methods address static images based on frame intervals and suffer from reduced accuracy because they ignore the diversity of different individual motions. In contrast, STG-NODE produces ODE-based dynamic graphs and properly aligns the key points of actions.

The development of skeleton-based action recognition methods has gone through three different stages.

1) Traditional feature engineering stage: In this early stage, researchers mainly relied on hand-designed feature extraction methods for processing skeleton data and performing action recognition. These methods often require expertise and experience to select and design features. The focus of this research concerns how to extract meaningful information from skeleton data. With the emergence of the machine learning era, researchers have begun to manually design skeleton data and shape them into pseudoimages^9–11^ or coordinate vector sequences^12,13^. The methods developed in this stage cannot fully express the complex information contained in skeleton data, and they have difficulty coping with the diversity and complexity of different actions. Therefore, the attained recognition accuracy is greatly limited, making it difficult for skeleton-based action recognition algorithms to be promoted or employed in wider application fields.

2) RNN and CNN stage: With the rise of deep learning technology, skeleton-based action recognition has undergone a revolutionary change. Deep learning models such as recurrent neural networks (RNNs) and convolutional neural networks (CNNs) are beginning to be employed for processing skeleton data. The focus of an RNN is to model the time series information contained in skeleton data to capture the time series characteristics of actions^14–16^. RNN models, such as long short-term memory (LSTM), can handle long-term dependencies, automatically extract key features, and map them to action categories. This capability enables them to achieve action recognition^17–19^. In contrast, the main goal of CNNs is to extract local features from skeleton data through convolution operations to identify key skeleton patterns. A CNN exhibits spatial invariance and can ignore the specific positions of joints. It gradually extracts hierarchical features through multilayer convolution and is finally combined with a classifier to achieve action recognition^20–22^. Nonetheless, the effectiveness of the abovementioned RNN and CNN methods in terms of recognizing actions from skeletal data is still limited. This limitation stems from the inability of these methods to openly represent the spatial relationships between joints, preventing neural networks from directly and proficiently capturing the collaborative spatial interactions between joints.

3) GCN stage: In recent years, the concept of graph convolutional networks (GCNs), as an extension of the convolution paradigm from images to graphs, has notably penetrated various fields^23–26^. The intrinsic graph structure of a non-Euclidean space is seamlessly coordinated with the intrinsic configuration of the human skeleton and is essentially a graph-like structure. Joints act as vertices and are connected to each other through edges that reflect the connections between bones in the human body. This complex architecture enables the description of dependencies in interconnected joints. The pioneering work^27^ concerning spatial-temporal graph convolutional networks (ST-GCNs), which encapsulate human skeleton data within graph frameworks, is particularly important. In this approach, a GCN is used for skeleton-based action recognition. This impetus has pushed GCN-based methods to the forefront of recognition tasks, cleverly capturing the subtleties of space and time by constructing spatiotemporal graphs. It is worth noting that this type of method demonstrates not only significant robustness but also commendable computational efficiency^14,28^.

Specifically, the AS-GCN model proposed by Li et al.^29^ successfully captures richer dependencies and more detailed patterns in actions. The model proposed by Peng et al.^30^ uses a neural architecture search (NAS) to explore the spatiotemporal correlations between nodes and employs multiple dynamic graph modules to construct a search space. However, most GCN variants, including the abovementioned models, ignore the issue of “intraindividual action differences”. For example, the same action performed by the same person at different times or locations will produce different action attributes and differences, and these attributes and differences can seriously affect the recognition accuracy of the utilized model. The AGC-LSTM model proposed by Si et al.^31^ successfully captures discriminative spatial configuration features and temporal dynamics and successfully explores the co-occurrence relationship between the spatial and temporal domains. Subsequently, Shi et al.^32^ proposed the MS-AAGCN model that uses a data-driven approach to increase its flexibility and generalization capabilities; the authors confirmed that the adaptive learning graph topology is more suitable for action recognition tasks than human-based graphs. The above approaches are all valid spatiotemporal network models, but they mainly consider short-range connections. However, the MST-GCN model proposed by Chen et al.^33^ proved that long-range dependencies are also important for action recognition. Compared with traditional deep neural networks, an ordinary GCN or a spatiotemporal GCN model significantly improves the resulting recognition accuracy. However, according to our analysis, the current mainstream models face at least the following two challenges. 1) The intraindividual differences among actions are neglected. The current GCN-based models often ignore the impact of intraindividual action differences on the accuracy achieved in skeleton-based action recognition tasks. 2) The susceptibility of the existing graph neural networks (GNNs) to oversmoothing reflects an inherent limitation of these networks. As the network layers deepen, all node representations tend to converge to a uniform value, which greatly affects the ability of the employed model to capture long-term dependencies, especially long-term temporal dependencies.

To this end, the spatial-temporal graph neural ODE network (STG-NODE) proposed herein integrates well-designed components to overcome these challenges. As shown in Figure 1, compared with the traditional methods, STG-NODE has excellent advantages in terms of accurately identifying key actions. First, its discretization layer with residual connections, inspired by^34^, can be viewed as a discretization of ODEs. Subsequently, a continuous graph neural network (CGNN)^35^ is derived to alleviate the oversmoothing problem. Taking advantage of this strategy, an ODE-temporal convolutional network (TCN) module is developed to enhance the temporal modeling ability of the model so that it can simulate long-term temporal dependencies. It is worth mentioning that the dynamics introduced by the ODE-TCN module improve the interpretability of the model in this task domain. Second, STG-NODE designs a semantic-based adjacency matrix for skeleton-based action recognition. This innovation is based on an elaborate data preprocessing pipeline, which includes skeleton alignment, semantic feature extraction, category labeling, and dynamic time warping (DTW)-based similarity computation steps, resulting in a semantic adjacency matrix. This unique approach significantly improves the flexibility of the STG-NODE model by effectively mitigating the impact of intraindividual action differences on the accuracy of the skeleton-based action recognition process, ultimately improving its performance in terms of recognizing complex human motions from skeletal data. To verify the superiority of our proposed model (i.e., STG-NODE), we conduct extensive experiments on two large datasets: the NTU RGB+D 60 dataset^13^ and the Kinetics Skeleton 400 dataset^36^. Our model achieves superior performance to that of the competing methods on both datasets.

The main contributions of our work are as follows.

- The main contribution of STG-NODE is the introduction of tensor-based ordinary differential equations for conducting skeleton-based action recognition. Specifically, this study designs an ODE-TCN module to enhance the temporal modeling capabilities of the model. This enhancement enables the model to effectively model long-term temporal dependencies, thereby improving its suitability for tasks involving complex temporal patterns. Notably, the dynamics introduced by the ODE-TCN module enhance the interpretability of the resulting model in the context of skeleton-based action recognition.
- A semantic adjacency matrix customized for skeleton-based action recognition is proposed. This innovation is based on a customized data preprocessing pipeline and a similarity calculation with DTW, ultimately creating a semantic-based semantic adjacency matrix. This unique approach enhances the flexibility of the model and, ultimately, its performance with respect to recognizing complex human motions from skeletal data.
- To validate the effectiveness of our proposed STG-NODE model, we conduct extensive experiments on two large datasets: NTU RGB+D 60 and Kinetics Skeleton 400. Our model consistently achieves superior performance on both datasets, demonstrating its ability to address the challenges encountered in skeleton-based action recognition tasks.

The remainder of this paper is organized as follows. Section “The proposed approach” first briefly describes the motivation of this paper, followed by detailed descriptions of the key components in the proposed STG-NODE model. Section “Experiments” verifies the effectiveness of our method through comparative and ablation experiments and analyses. Finally, we conclude the paper in Section “Conclusion”.

## The proposed approach

### Motivation

Repeatedly performing the same action introduces diversity in skeletal data due to variations in physiological characteristics and action execution conditions. This phenomenon, which is known as intraindividual action differences, affects recognition accuracy. Furthermore, the importance of long-term temporal dependencies is evident in action recognition tasks since actions are coherent and sequential in nature. Neglecting temporal dependencies may lead to information losses and limited recognition accuracy.

These challenges drive our innovative approach. First, the motivation behind the introduction of tensor-based ODEs stems from the need to capture and model the long-term temporal dependencies inherent in skeleton data. Theoretically, the dynamics resulting from ODEs fit the inherent coherence properties of skeleton-based action recognition tasks. To this end, the ODE-TCN module is introduced to enhance the temporal modeling capabilities of our model and simultaneously inject dynamics to better simulate the temporal attributes of real actions, allowing the model to more accurately capture the dynamic changes exhibited by action sequences. Second, the motivation behind introducing semantic-based category similarity matrices is to address the intraindividual action differences encountered in skeleton-based action recognition scenarios. Theoretically, we plan to obtain semantic action features through specific operations and then calculate the target matrix. To this end, by carefully designing a data preprocessing strategy, including alignment, semantic feature extraction, category labeling, and DTW-based similarity calculations, this study creates a novel semantic adjacency matrix. This matrix is expected to enhance the adaptability of the developed model by effectively mitigating the effect of intraindividual action differences, thereby improving the accuracy of skeleton-based action recognition. These efforts ultimately lead to the development of the STG-NODE model.

**Figure 2:**
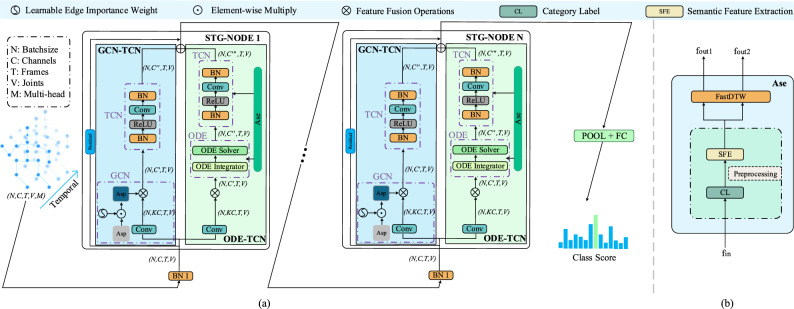
(**a**) Basic network framework. The POOL-FC layer and the final class score component form the output module. Asp represents the spatial adjacency matrix; Ase represents the semantic adjacency matrix. (**b**) The details of Ase. fin denotes the original data; fout1 and fout2 are the category similarity matrices.

### Model framework

Figure 2(a) shows the basic framework of our proposed STG-NODE model. It mainly consists of three parts: an ordinary differential equation-temporal convolutional network (ODE-TCN) module, a graph convolutional network-temporal convolutional network (GCN-TCN) module and an output module. The ODE-TCN module is composed of an integrator, a solver and a temporal convolutional network connected in series. The integrator is implemented with an integral function to generate a solution function, which is obtained by integrating the input data in the temporal dimension. The solver is implemented with an ordinary differential equation solver based on the numerical solution with respect to the time characteristics of the solution function so that the model can effectively model long-term time dependencies. The GCN-TCN module is composed of a graph convolutional network and a temporal convolutional network in series; this module empowers the model by ensuring that it comprehensively considers and leverages the joint relationships and spatial structures contained within skeleton data. This leads to an improved understanding and analysis of the spatial features and relationships in human body movements, ultimately resulting in enhanced model performance. Functionality of the Output Module: This module meticulously consolidates and summarizes the features acquired from skeleton data. This process enables the amalgamation of skeleton features into higher-level representations, effectively capturing the abstract characteristics of various actions. Subsequently, these refined features are mapped to various potential action categories, yielding probability distributions for each category. Furthermore, both the ODE-TCN and GCN-TCN modules conduct feature extraction in parallel across different layers. Following feature fusion, these modules seamlessly feed the features into the subsequent parallel structure. Ultimately, the amalgamated features are fed into the output module, enabling the model to perform action classification and accurately determine the action category to which the input data belong. To better present the details of the ODE-TCN module, we show the Ase submodule separately in Figure 2(b).

### Adjacency matrix construction

In our model, we use two types of adjacency matrices. Drawing inspiration from the ST-GCN^27^, the spatial adjacency matrix is formulated as follows:

1 $$\begin{aligned} A_{ij}^{sp}= \left\{ \begin{matrix} A_{ij}\cdot D_{ij},\ if\ A_{ij}^{hop}\ne inf\\ 0,\ otherwise \end{matrix}\right. \end{aligned}$$

A is an adjacency matrix, D is a diagonal matrix whose diagonal elements are node degrees, $A^{hop}$ is the shortest number of hops between nodes, and inf means that the corresponding nodes are not reachable.

**Figure 3:**
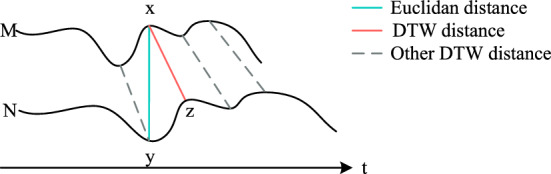
An example of the difference between the Euclidean distance and the FastDTW distance.

In addition, it is crucial to consider the impact of intraindividual action differences on the accuracy of skeleton-based action recognition tasks. For example, the same person performing the same action at different times or locations produces different action properties and differences that cannot be revealed in a spatial graph. To capture the above semantic variability, we use the FastDTW^37^ algorithm to calculate the joint nodes of a human body action based on the node characteristics (all node coordinates within a period of time), thereby constructing a semantic adjacency matrix. This semantic adjacency matrix quantifies the degrees and strengths of the correlations between joint points in human movements through an algorithm, thereby extracting semantic information. By providing semantic information concerning action execution, the semantic adjacency matrix enables the model to better understand the intrinsic correlations and meanings of actions. As shown in Figure 3, with the FastDTW algorithm, point a of a series M is correlated with point z of another series N but not with point y of series N. Specifically, given two time series $M = (m_{1},\ m_{2},\cdots ,\ m_{k})$ and $N = (n_{1}, n_{2},\cdots , n_{l})$, FastDTW is a dynamic programming algorithm that is defined as follows:

2 $$\begin{aligned} \small D(i,\ j)=dist(m_{i},\ n_{j})+min(D(i-1,\ j),\ D(i,\ j-1),\ \varepsilon *D(i-1,\ j-1)) \end{aligned}$$

Where $D(i,\ j)$ denotes the shortest distance between subseries $M=(m_{1},\ m_{2},\cdots ,\ m_{i})$ and $N=(n_{1},\ n_{2},\cdots ,\ n_{j})$, with $dist(m_{i},\ n_{j})$ representing a distance metric such as the absolute distance measure. Consequently, $FastDTW(M,\ N)=D(k,\ l)$ signifies the ultimate distance between *M* and *N*, providing a more accurate assessment of the similarity between two time series than that provided by the Euclidean distance. The value of the multiplier $\epsilon$ ranges from 0 $< \epsilon \leqslant$ 1. By adjusting the value of $\epsilon$, the approximation degree of the FastDTW algorithm can be controlled, which reduces its computational complexity to a certain extent and improves the computational speed of the model.

Accordingly, we define the semantic adjacency matrix through the FastDTW distance as follows:

3 $$\begin{aligned} A_{ij}^{se}=\left\{ \begin{matrix} 1,&{}FastDTW(X^{i},\ X^{j})<\varepsilon \\ 0,&{}otherwise \end{matrix}\right. \end{aligned}$$

where $X^{i}$ represents the time series of node *i*, and $\epsilon$ controls the sparsity level of the adjacency matrix.

### Customized ODE integrator and solver

GCNs update node embeddings by aggregating features derived from both the nodes themselves and their neighbors using a graph convolution operation. The conventional form of this convolution operation can be expressed as follows:

4 $$\begin{aligned} f_{out}=GCN(f_{in})=\alpha (f_{in}{\hat{A}}W) \end{aligned}$$

where $f_{in}\in {\mathbb {R}}^{N\times C}$ denotes the input of the previous graph convolution layer, ${\hat{A}}\in {\mathbb {R}}^{N\times N}$ is the normalized adjacency matrix, and $W\in {\mathbb {R}}^{C\times {C}'}$ is a learnable parameter matrix that models the interactions among different features. However, such GCNs have been shown to suffer from the issue of oversmoothing as the networks become deeper^38,39^, which significantly restricts their ability to model long-range dependencies. In response to this limitation, we introduce our novel STG-NODE block.

To allow interactions between the adjacency matrices and modules, we are inspired by the success of the CGNN^35^ and consider a more powerful discrete dynamic function:

5 $$\begin{aligned} f_{out}=f_{in}\times _{1} {\hat{A}}\times _{2} Z\times _{3} W+h_{0} \end{aligned}$$

where $f_{in}\in {\mathbb {R}}^{N\times T\times F}$ is a space-time tensor that represents the hidden embedding of the examined node in the previous layer, $\times _{i}$ denotes the tensor matrix multiplication operation executed on mode *i*, ${\hat{A}}$ is the regularized adjacency matrix, *Z* is the temporal transformation matrix, *W* is the feature transformation matrix, and $h_{0}$ denotes the initial input of the GCN, which can be acquired through another neural network. Motivated by the CGNN, a restart distribution $H_{0}$ is used to alleviate the oversmoothing problem.

Although the residual structure shown in Eq. 5 is powerful, training it can be challenging due to the large number of parameters involved. Therefore, our goal is to extend the discrete formulation to a continuous expression in the skeleton-based action recognition domain. To effectively convert the residual structure into an ODE structure, we follow the successful practices of previous researchers, such as the series of practices adopted in^40^.

Specifically, the continuous expression of Eq. 5 is shown as follows:

6 $$\begin{aligned} \frac{df(t)}{dt}=f(t)\times _{1} ({\hat{A}}-I)+f(t)\times _{2} (Z-I)+f(t)\times _{3} (W-I)+H_{0} \end{aligned}$$

Finally, we draw inspiration from neural ODEs^34^ and introduce our STG-NODE framework. The continuous form of the hidden representation is as follows:

7 $$\begin{aligned} f(t)=ODESolver(\frac{df(t)}{dt},h_{0},t) \end{aligned}$$

Runge-Kutta solvers are generally more stable than Euler solvers, which is critical for accurately capturing the subtle changes and characteristics of action sequences. In addition, a Runge-Kutta solver has higher accuracy when processing action sequences with nonlinear characteristics and rapid changes and can more accurately capture details and important features from actions. Based on these considerations, we choose the Runge-Kutta method as the ODE solver in our model.

### STG-NODE module and performance analysis

The preceding sections have provided a detailed exposition of the key components contained within STG-NODE. This section operates from a macro perspective, delineating the holistic STG-NODE module. As illustrated in Figure 1, the model adopts a serial-parallel structure comprising an ODE-TCN block and a GCN-TCN block. This inventive architecture not only facilitates the seamless amalgamation of spatiotemporal information but also harnesses the inherent strengths of ODEs, thereby enhancing the precision achieved in skeleton-based action recognition tasks.

On the one hand, STG-NODE presents a plethora of advantages over traditional GCNs and TCNs, significantly bolstering the foundational aspects of skeletal action recognition models. ODEs fundamentally capture dynamic behaviors by modeling state evolution trends over consecutive time intervals. This property impeccably aligns with the nuanced nature of human motion, enabling the model to discern the subtle temporal intricacies contained within skeletal data. Furthermore, the ODE-based framework exhibits superior generalization capabilities to those of the traditional methods and adeptly handles irregularly sampled or missing data points within the input skeleton sequence. Its rich feature reservoir and adept module combinations substantially augment the ability of the model to unravel intricate the correlations inherent in skeletal actions. However, compared to models that lack DTW integration (e.g.,^27,41^), the ODE-TCN module utilizes FastDTW to compute the semantic adjacency matrix. This matrix enables the ODE-TCN module to focus more on nodes with greater relevance when propagating information in the temporal dimension during the training process, thereby capturing key frames in actions with varying lengths. This allows the model to better adapt to situations where the execution speed is faster or slower than the action features are learned. Ultimately, this mechanism allows STG-NODE to mitigate the impact of intraindividual action differences.

In summary, the STG-NODE model has the advantages of ODEs and DTW enhancement. In theory, this comprehensive advantage makes the STG-NODE model significantly better than the traditional models. This enhancement is reflected in its ability to effectively capture the spatiotemporal complexity of skeletal actions, resulting in significant performance advantages in action recognition tasks.

### Training loss expression

The cross-entropy loss is chosen as the loss function, as it is well suited for addressing multiclassification tasks and exhibits strong sensitivity to variations in predicted probability distributions. This property encourages the model to prioritize the correct category. The formulation of the cross-entropy loss is outlined below:

8 $$\begin{aligned} L(y,t)=-\sum _{c=1}^{C}t_{c}log(y_{c}) \end{aligned}$$

where *C* is the number of categories, $t_{c}$ is the value of the *c*-th category in the real labels, $y_{c}$ is the predicted probability output by the model for the *c*-th category, *y* is the output of the model, indicating the probability predicted by the model for each category, *t* is the real label, only one element is 1, and the others are 0.

The goal of the loss function is to guide the optimization process of the model parameters by minimizing the difference between the predicted probability of each category and the one-hot encoding of the actual corresponding label, thereby enabling the model to more accurately predict action categories.

## Experiments

In this section, an extensive performance evaluation of the devised STG-NODE model is implemented across two expansive datasets: NTU RGB+D 60^13^ and Kinetics Skeleton 400^36^. Given the relatively modest size of the NTU RGB+D 60 dataset, a meticulous ablation study is conducted to ascertain the efficacy of the enhancements incorporated into the model. Subsequently, a comprehensive comparative analysis is performed, benchmarking our STG-NODE model against other approaches. This multifaceted evaluation, spanning two datasets (two completely different benchmarks plus two different indicator scales), serves to corroborate both the broad applicability and the precision of our proposed framework in terms of achieving definitive recognition outcomes.

### Datasets

The **NTU RGB+D 60** dataset, which contains an extensive collection of 56,000 action clips classified into 60 different action categories, is of crucial importance in the field of 3D human action recognition. This comprehensive range covers a variety of action types, from the nuances of everyday behaviors to health-related routines and complex two-person interactions. These captured segments were recorded from the perspective of three synchronized camera angles within the controlled confines of a laboratory environment. These visual narratives reveal the complex spatial coordinates of joints (X, Y, Z) in 3D with the help of the discriminative capabilities provided by the Kinect depth sensor.

The evaluation paradigm for this dataset is carefully constructed around two strong paradigms: cross-subject (X-Sub) and cross-view (X-View) protocols. Under the X-Sub benchmark, the partitioning strategy is based on individual IDs and ultimately allocates 40,320 samples for fine-grained training and an additional 16,560 samples for rigorous testing. At the same time, the X-View framework utilizes perspectives derived from different camera angles to form similar partitioning patterns. In this configuration, a selective subset of 18,960 samples from camera 1 is reserved for exhaustive testing purposes, while a large repository of 37,920 samples acquired from cameras 2 and 3 strongly supports the comprehensive training scheme.

The **Kinetics Skeleton 400** dataset is a comprehensive compilation of approximately 300,000 video clips that were carefully curated from various sources on YouTube. This massive dataset contains 400 different human action categories, covering a wide range of scenarios from everyday activities to dynamic motion scenarios and complex interactions with complex actions. Notably, each video clip in the Kinetics Skeleton 400 dataset maintains a consistent temporal structure with an average duration of approximately 10 seconds. The clips were captured at a standard frame rate of 30 frames/second, yielding a total of 300 frames.

Each frame in these clips is meticulously analyzed, during which up to two joints with the highest average confidence levels are selected. This rigorous process culminates in the precise definition of 18 joints for each bone structure, each of which characterized by its 2D coordinates and corresponding confidence. The resulting skeletal representation provides the basis for a comprehensive action analysis.

Furthermore, in the context of the Kinetics Skeleton 400 experiment, we evaluate the achieved recognition performance according to the top-1 and top-5 classification accuracy metrics, which evaluate the determinism and robustness of the tested model. The estimated 2D joint positions generated by the OpenPose pose estimation framework^42^ provided by the ST-GCN are used as inputs. This choice ensures that the experiments are performed on a consistent and reputable basis, allowing for a robust and accurate analysis of dataset-rich human behavioral dynamics.

### Experimental settings

The experiments are conducted on a Linux server equipped with an Intel(R) Xeon(R) Silver 4316 CPU running at 2.3 GHz and four NVIDIA TESLA A100 GPUs. Adhering to the foundational framework of the ST-GCN, STG-NODE follows a similar structural setup. The architecture encompasses 10 STG-NODE blocks within the STG-NODE model, with each block consisting of one ODE-TCN and one GCN-TCN. For the semantic adjacency matrix, precise calibration is achieved by setting the thresholds $\sigma$ and $\epsilon$ to 0.1 and 0.6, respectively.

Stochastic gradient descent (SGD) with Nesterov momentum (0.9) is applied as the optimization strategy with a learning rate of 0.15, and it is accompanied by a decay ratio of 0.0001. The cross-entropy loss function is chosen for gradient backpropagation. This process is executed using a batch size of 64, spanning a training period that extends to 80 iterations. Notably, the experimental setup applies several supplementary preprocessing strategies to each dataset. Initially, the coordinate information of the samples is extracted through the utilization of a dedicated skeleton sequence visualization tool, as provided in^27^. Subsequently, the extracted data are carefully processed using the DTW algorithm, and finally, a customized semantic adjacency matrix is derived. This strategic preprocessing procedure augments the discernment capabilities of the model and contributes to the overall efficacy of the experimental analysis.

### Comparison

To evaluate the performance of STG-NODE, we compare our proposed STG-NODE model with other skeleton-based action recognition methods on the Kinetics Skeleton 400 dataset and the NTU RGB+D 60 dataset. The comparison results are shown in Table 1, which shows that our model has strong performance advantages on both datasets.

**Table 1:** Accuracy comparisons between our proposed STG-NODE model and other methods on NTU RGB+D 60 and Kinetics Skeleton 400. Significant values are in bold.

Method	X-Sub	X-View	Kinetics Top1	Kinetics Top5	Years
Deep LSTM^13^	60.7	67.3	16.4	35.3	2016
TCN^11^	74.3	83.1	20.3	40.0	2017
ST-GCN^27^	81.5	88.3	30.7	52.8	2018
DS-LSTN^43^	75.5	84.2	-	-	2020
STD+RGB-DI^44^	79.4	84.1	-	-	2020
GFNet^45^	82.0	89.9	-	-	2020
STA^46^	72.4	79.7	-	-	2021
CNN+LSTM^47^	81.9	88.7	-	-	2021
PoT2I^48^	83.9	90.3	-	-	2021
C-CNN+HTLN^49^	83.5	86.8	-	-	2022
Custom ST-GCN^41^	82.7	90.2	32.3	54.5	2023
STG-NODE (ours)	**84**.**0**	**91**.**1**	**32**.**6**	**55**.**0**	2023

Specifically, our model produces remarkable recognition results on the Kinetics Skeleton 400 and NTU RGB+D 60 datasets, achieving the best performance. However, when delving into the nuances of the Kinetics dataset, the recognition accuracy improvement achieved over the method of^41^ is not sufficiently large. A plausible explanation for this phenomenon can be attributed to the composition of the Kinetics Skeleton 400 test set. This dataset contains individuals whose behavioral styles or unique characteristics are not fully represented within the scope of the training set. The emergence of this new individual variability poses urgent challenges and may require the inclusion of additional data to enhance the generalizability of the model. Thus, it becomes evident that our model yearns for a more substantial sample size to adeptly assimilate and adapt to the diverse intricacies of different individuals, thereby elevating its performance on the Kinetics dataset.

Furthermore, notably, it performs well on the NTU RGB+D dataset, surpassing the benchmarks set by models such as that of^41^. This serves as a compelling testament to the exceptional prowess of our model with respect to addressing action recognition tasks from varying viewpoints. The undeniable significance of this capacity becomes even more pronounced in real-world applications.

**Figure 4:**
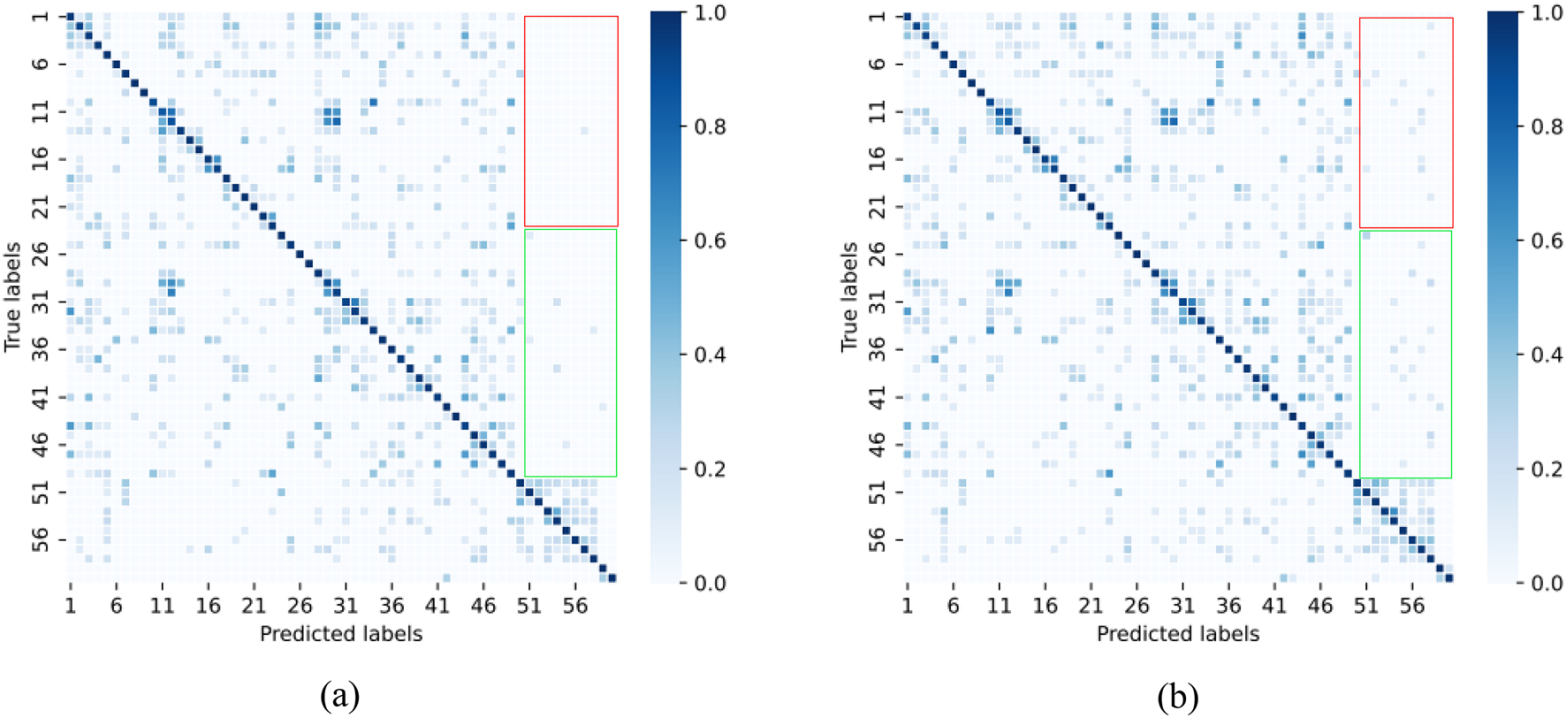
Confusion matrices produced for the NTU RGB+D 60 dataset. (**a**) shows the experimental results of STG-NODE; (**b**) presents the experimental result of the ST-GCN.

To more clearly highlight the advantages of STG-NODE over the existing models, we select some action categories with obvious coherence differences for comparison. Generally, people have a better memory and understanding of common action sequences and therefore perform more naturally and coherently when performing such actions. We call these action categories “strong-coherence action categories”, as shown in the red box in Figure 4, including drinking water (label: 1), sitting down (label: 8), standing (label: 9), etc. Conversely, some actions may feel confusing or unnatural, with less coherence. We call these action categories “weakly coherent action categories”, as shown in the green box in Figure 4, including typing on a keyboard (label: 30), experiencing back pain (label: 46) and vomiting (label: 48), etc. In Figure 4, we clearly observe that in the weakly coherent action category, STG-NODE has a smaller performance improvement over the ST-GCN (dark areas remain in the green box). However, in the strongly coherent action category, STG-NODE yields significantly improved performance over that of the ST-GCN model (the dark areas in the red box have basically disappeared).

The two datasets used in the experiments exhibit distinctly disparate properties. While the Kinetic dataset employs a 2D skeleton detected by a deep neural network as the input, the NTU RGB+D 60 dataset employs data from a Kinect depth sensor. Further differentiating the two datasets, NTU RGB+D 60 employs a stationary camera, whereas the Kinetics dataset often captures videos using handheld devices, thus introducing significant camera motion. The noteworthy efficacy exhibited by the proposed STG-NODE model across both datasets underscores the prowess of our spatiotemporal dependency modeling approach. This accomplishment can be attributed to two key factors.

- Leveraging the tensor-based ODE framework significantly augments the temporal modeling ability of the model. Simultaneously, the dynamics introduced by the ODE can be construed as the evolutionary journey of an action sequence, thereby providing insights into the rationale used by the model for action recognition.
- Employing a strategic approach, the DTW algorithm serves as a conduit that introduces the semantic adjacency matrix. This augmentation bolsters the semantic acumen of the model and adeptly mitigates the influence of individual intraindividual action discrepancies on the precision achieved in skeleton-based action recognition tasks.

### Ablation study

Ablation experiments concerning action recognition are performed on the NTU RGB+D 60 dataset and the Kinetics Skeleton 400 dataset to examine the effectiveness of the proposed components in the above STG-NODE model. Then, different learning rates are set for a verification implemented on the X-Sub benchmark and X-View benchmark to achieve the best recognition accuracy.

**Evaluation of the effectiveness of each STG-NODE module:** To determine the necessity and effectiveness of the individual modules in the STG-NODE model, a system analysis is performed by iteratively omitting certain modules from the original architecture and subsequently comparing the performances of the ablated versions. Two different variants of the STG-NODE model are designed for this purpose.

1. STG-Semantic: In this model, a semantic adjacency matrix is constructed based on the semantic similarity of the target skeleton. However, the ODE solver is replaced by a regular GCN to verify the effectiveness of the ODE structure in terms of capturing long-range dependencies.

2. STG-ODE*: This model contains ODE modules but does not involve the creation of specialized adjacency matrices. This omission is intended to identify the necessity of introducing a semantic adjacency matrix.

**Table 2:** Comparisons between the accuracy (%) of our model and that of each variant on the NTU RGB+D 60 dataset and the Kinetics Skeleton 400 dataset.

Method	X-Sub	X-view	Kinetics Top1	Kinetics Top5
STG-semantic	80.8	89.0	30.8	53.1
STG-ODE*	81.9	90.1	31.4	53.7
STG-NODE	84.0	91.1	32.6	55.0

The results are shown in Table 2, which shows that the accuracy and efficiency of a model adding any module exceed those of the baseline model. It is worth noting that the best performance is achieved when all modules are integrated. This synergy leads to significant improvements in the accuracies achieved on the X-View and X-Sub benchmarks of the NTU RGB+D 60 dataset (2.1% and 3.2%, respectively), while improving the top-1 and top-5 accuracies attained on the Kinetics Skeleton 400 dataset by up to 1.8% and 1.9%, respectively.

**Figure 5:**
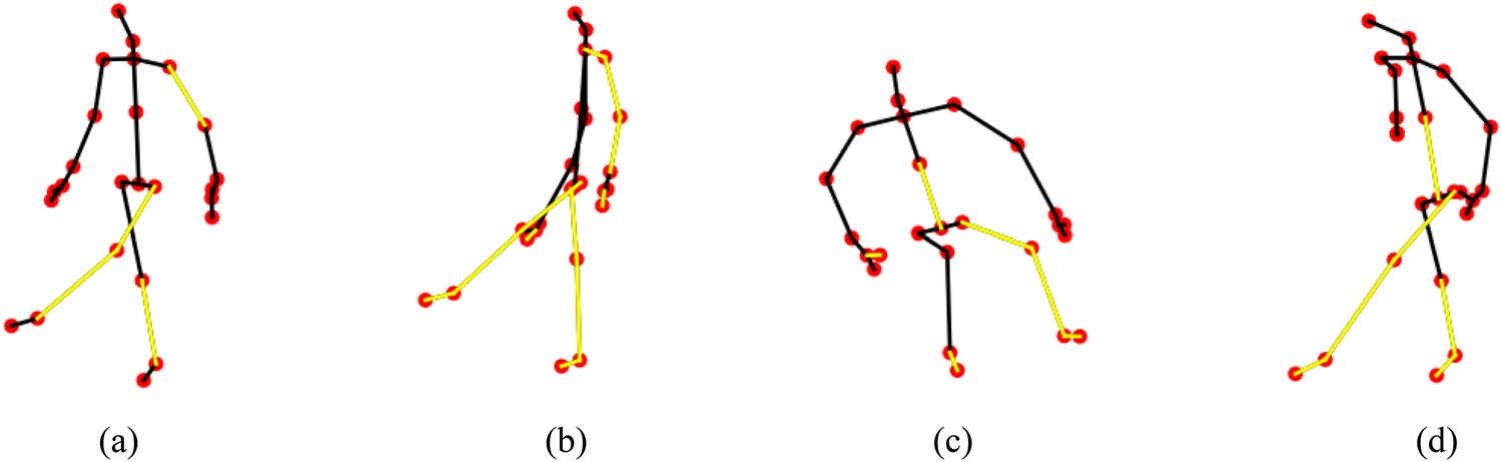
Visualization of kicking actions. (**a**) is the first action of person No. 1, (**b**) is the second action of person No. 1, (**c**) is the first action of person No. 2, and (**d**) is the second action of person No. 2. The two actions occur at different angles, heights and distances.

These results underscore the significant performance improvement provided by our STG-NODE architecture due to its innovative temporal modeling approach and specialized semantic adjacency matrix. In addition, to more clearly demonstrate the effect of integrating the semantic adjacency matrix into the ODE-TCN module, we draw action visualization diagrams of different individuals performing the same action (such as kicking something). As shown in Figure 5, the yellow highlighted part represents the edge composed of joint points with higher correlations when executing the action. When the model encounters different individuals performing the same action at different speeds, it focuses on these parts to help mitigate the impact of intraindividual action differences on the accuracy of skeleton-based action recognition.

**Figure 6:**
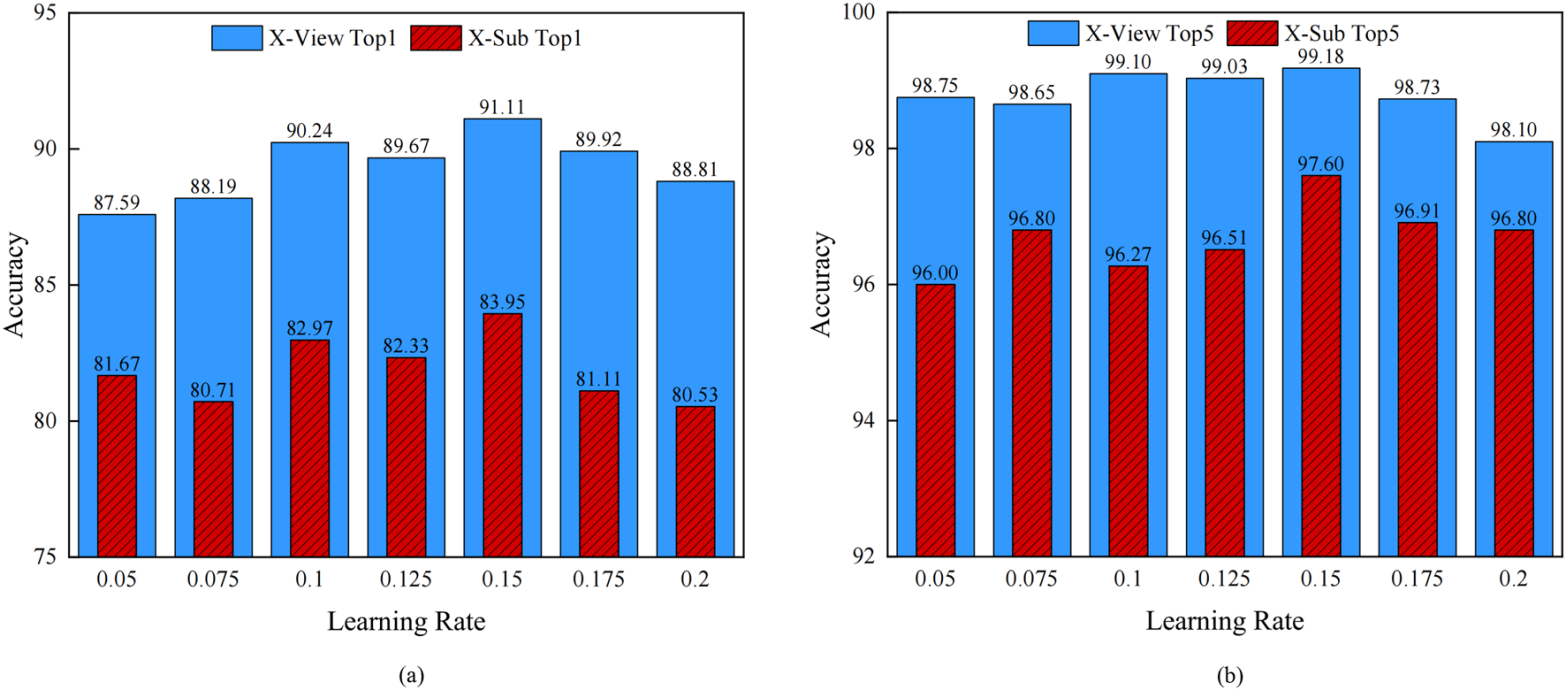
Recognition accuracy fluctuations observed in two accuracy evaluations conducted on the NTU RGB+D dataset with different learning rates.

**Selection of the Optimal Learning Rate for STG-NODE:** We conduct a comprehensive evaluation of the accuracy achieved by STG-NODE across 7 distinct learning rates. Figure 6 (b) compares the top-5 accuracy results obtained on the two benchmarks, and Figure 6 (a) compares the top-1 accuracy results obtained on the two benchmarks. Notably, as depicted in the figure, the experimental accuracy peaks when the learning rate is set to 0.15.

## Conclusion

Many efforts have been made to address the complex challenge of action recognition. However, little attention has been given to solving the difficult problem of extracting long-range dependencies without succumbing to the oversmoothing problem that is inherent in GCN-related architectures. This paper presents a groundbreaking ODE-based spatiotemporal forecasting model called STG-NODE. To the best of our knowledge, this is the first attempt to link continuous differential equations to node representations for developing skeleton-based action recognition networks, and STG-NODE provides the ability to shape deeper architectures and exploit a wider range of dependencies than can other methods. Furthermore, the incorporation of a customized semantic adjacency matrix greatly improves the efficiency of the model. The performance achieved by STG-NODE in four challenging tests (two benchmarks plus two metrics) is better than that of many existing methods. In future research, we will delve into the extraction of complex local features from skeletons and consider further exploiting a graph structure to capture the relationships between different parts of an input sequence, such as the interaction dependencies between different body parts in human activity recognition tasks.

## Data Availability

All data included in this study are available upon request by contact with the corresponding author.
